# *trans*-Bis(5-carb­oxy-2-methyl-1*H*-imidazole-4-carboxyl­ato-κ^2^ *N* ^3^,*O* ^4^)copper(II)

**DOI:** 10.1107/S1600536809016791

**Published:** 2009-05-14

**Authors:** Xin Guo, Yi Liang Li, Ju Xian Wang, Yu Cheng Wang

**Affiliations:** a Institute of Medicinal Biotechnology, Chinese Academy of Medical Sciences, and Peking Union Medical College, Beijing 100050, People’s Republic of China

## Abstract

In the title compound, [Cu(C_6_H_5_N_2_O_4_)_2_], the copper(II) atom lies on an inversion centre and is in an N_2_O_2_ four-coordinate environment with a nearly regular square-planar geometry. An extended network of intra­molecular O—H⋯O and inter­molecular N—H⋯O and C—H⋯O hydrogen bonds stabil­izes the crystal structure.

## Related literature

For the synthesis and crystal structure of metal complexes with *N*-heterocyclic carboxylic acids, see: Nie *et al.* (2007 ▶); Liang *et al.* (2002 ▶); Net *et al.* (1989 ▶); Zeng *et al.* (2008 ▶).
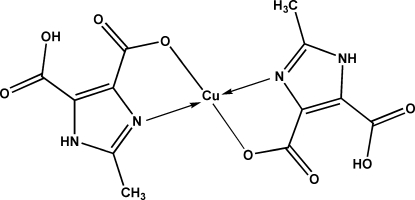


## Experimental

### 

#### Crystal data

[Cu(C_6_H_5_N_2_O_4_)_2_]
*M* *_r_* = 401.78
Monoclinic, 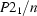

*a* = 7.3780 (17) Å
*b* = 7.575 (2) Å
*c* = 12.863 (3) Å
β = 101.287 (13)°
*V* = 705.0 (3) Å^3^
*Z* = 2
Mo *K*α radiation
μ = 1.61 mm^−1^
*T* = 292 K
0.30 × 0.25 × 0.20 mm

#### Data collection

Rigaku SCXmini diffractometer
Absorption correction: multi-scan (*CrystalClear*; Rigaku, 2005 ▶) *T*_min_ = 0.638, *T*_max_ = 0.727
5198 measured reflections
1609 independent reflections
1472 reflections with *I* > 2σ(*I*)
*R*_int_ = 0.019

#### Refinement

*R*[*F* ^2^ > 2σ(*F* ^2^)] = 0.030
*wR*(*F* ^2^) = 0.084
*S* = 1.07
1609 reflections
116 parameters
H-atom parameters constrained
Δρ_max_ = 0.28 e Å^−3^
Δρ_min_ = −0.66 e Å^−3^

### 

Data collection: *CrystalClear* (Rigaku, 2005 ▶); cell refinement: *CrystalClear*; data reduction: *CrystalClear*; program(s) used to solve structure: *SHELXS97* (Sheldrick, 2008 ▶); program(s) used to refine structure: *SHELXL97* (Sheldrick, 2008 ▶); molecular graphics: *SHELXTL/PC* (Sheldrick, 2008 ▶); software used to prepare material for publication: *SHELXTL/PC*.

## Supplementary Material

Crystal structure: contains datablocks I, New_Global_Publ_Block. DOI: 10.1107/S1600536809016791/rz2318sup1.cif

Structure factors: contains datablocks I. DOI: 10.1107/S1600536809016791/rz2318Isup2.hkl

Additional supplementary materials:  crystallographic information; 3D view; checkCIF report

## Figures and Tables

**Table 1:** Hydrogen-bond geometry (Å, °)

*D*—H⋯*A*	*D*—H	H⋯*A*	*D*⋯*A*	*D*—H⋯*A*
N2—H2*A*⋯O2^i^	0.86	2.01	2.822 (2)	157
C1—H1*A*⋯O4^ii^	0.96	2.49	3.217 (2)	132
C1—H1*B*⋯O3^iii^	0.96	2.58	3.285 (3)	130
C1—H1*C*⋯O1^iv^	0.96	2.43	3.258 (3)	144
O3—H3*A*⋯O2	0.91	1.67	2.576 (2)	171

Symmetry codes: (i) 
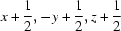
; (ii) 
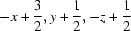
; (iii) 
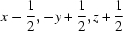
; (iv) 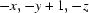.

**Fig. 1.**
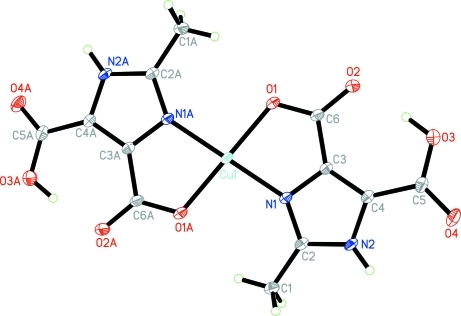
The molecular structure of the title compound, showing the atomic numbering scheme. Displacement ellipsoids are drawn at the 30% probability level. Atoms labelled with suffix A are generated by the symmetry operation (-x,1 - y,-z).

**Fig. 2.**
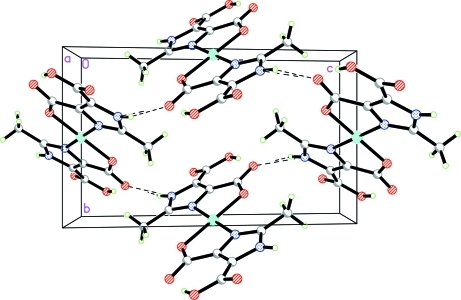
Packing diagram of the title compound viewed along the a axis. Intermolecular hydrogen bonds are shown as dashed lines.

## supplementary crystallographic information

### Comment

The study of metal complexes with *N*-heterocyclic carboxylic acids has been given considerable attention ((Nie *et al.*, 2007; Liang *et al.*, 2002; Net *et al.*, 1989; Zeng *et al.*, 2008). In this paper, we report on the synthesis and structure of the title compound, which was obtained by the hydrothermal reaction of CuCl_2 _with 2-methyl-1*H*-imidazole-4,5-dicarboxylic acid.

Figure 1 shows the monomeric complex molecule along with the atom-labelling scheme. The copper(II) metal lies on an inversion centre and is in an N_2_O_2_ four-coordinate environment with a regular square-planar geometry. The Cu—O distance is 1.9633 (15) Å and the Cu—N distance is 1.9830 (14) Å. The five-membered chelating ring assumes an approximately planar conformation (maximum deviation -0.033 (1) Å for atom N1). The crystal structure is stabilized by an intramolecular O—H···O hydrogen bond, and by intermolecular N—H···O and C—H···O hydrogen interactions (Table 1), forming an extended three-dimensional network (Fig. 2).

### Experimental

Blue single crystals of title compound were obtained by hydrothermal treatment of CuCl_2_ (1 mmol), 2-methyl-1*H*-imidazole-4,5-dicarboxylic acid (1 mmol) and water (5 ml) over 2 days at 388 K. Yield: 61% (based on CuCl_2_).

### Refinement

The hydroxyl H atom was located from a difference Fourier map but not refined [*U*_iso_(H)=1.5*U*_eq_(O)]. All other H atoms were placed at calculated positions and refined as riding, with C—H = 0.96 Å, N—H = 0.86 Å, and with *U*_iso_(H)=1.5*U*_eq_(C) and *U*_iso_(H)=1.2*U*_eq_(N)

### Crystal data

**Table:**

[Cu(C_6_H_5_N_2_O_4_)_2_]	*F*(000) = 406
*M**_r_* = 401.78	*D*_x_ = 1.893 Mg m^−^^3^
Monoclinic, *P*2_1_/*n*	Mo *K*α radiation, λ = 0.71073 Å
Hall symbol: -P 2yn	Cell parameters from 2111 reflections
*a* = 7.3780 (17) Å	θ = 3.1–27.5°
*b* = 7.575 (2) Å	µ = 1.61 mm^−^^1^
*c* = 12.863 (3) Å	*T* = 292 K
β = 101.287 (13)°	Prism, blue
*V* = 705.0 (3) Å^3^	0.30 × 0.25 × 0.20 mm
*Z* = 2	

### Data collection

**Table:**

Rigaku SCXmini diffractometer	1609 independent reflections
Radiation source: fine-focus sealed tube	1472 reflections with *I* > 2σ(*I*)
graphite	*R*_int_ = 0.019
Detector resolution: 13.6612 pixels mm^-1^	θ_max_ = 27.5°, θ_min_ = 3.1°
ω scans	*h* = −9→9
Absorption correction: multi-scan (*CrystalClear*; Rigaku, 2005)	*k* = −9→8
*T*_min_ = 0.638, *T*_max_ = 0.727	*l* = −16→16
5198 measured reflections	

### Refinement

**Table:**

Refinement on *F*^2^	Primary atom site location: structure-invariant direct methods
Least-squares matrix: full	Secondary atom site location: difference Fourier map
*R*[*F*^2^ > 2σ(*F*^2^)] = 0.030	Hydrogen site location: inferred from neighbouring sites
*wR*(*F*^2^) = 0.084	H-atom parameters constrained
*S* = 1.07	*w* = 1/[σ^2^(*F*_o_^2^) + (0.0506*P*)^2^ + 0.3299*P*] where *P* = (*F*_o_^2^ + 2*F*_c_^2^)/3
1609 reflections	(Δ/σ)_max_ < 0.001
116 parameters	Δρ_max_ = 0.28 e Å^−^^3^
0 restraints	Δρ_min_ = −0.66 e Å^−^^3^

### Special details

**Table:**

Geometry. All e.s.d.'s (except the e.s.d. in the dihedral angle between two l.s. planes) are estimated using the full covariance matrix. The cell e.s.d.'s are taken into account individually in the estimation of e.s.d.'s in distances, angles and torsion angles; correlations between e.s.d.'s in cell parameters are only used when they are defined by crystal symmetry. An approximate (isotropic) treatment of cell e.s.d.'s is used for estimating e.s.d.'s involving l.s. planes.
Refinement. Refinement of *F*^2^ against ALL reflections. The weighted *R*-factor *wR* and goodness of fit *S* are based on *F*^2^, conventional *R*-factors *R* are based on *F*, with *F* set to zero for negative *F*^2^. The threshold expression of *F*^2^ > σ(*F*^2^) is used only for calculating *R*-factors(gt) *etc*. and is not relevant to the choice of reflections for refinement. *R*-factors based on *F*^2^ are statistically about twice as large as those based on *F*, and *R*- factors based on ALL data will be even larger.

### Fractional atomic coordinates and isotropic or equivalent isotropic displacement parameters (Å^2^)

**Table:**

	*x*	*y*	*z*	*U*_iso_*/*U*_eq_	
Cu1	0.0000	0.5000	0.0000	0.02591 (14)	
C1	0.3363 (3)	0.5580 (3)	0.26068 (15)	0.0322 (4)	
H1A	0.4201	0.6562	0.2725	0.048*	
H1B	0.3575	0.4828	0.3219	0.048*	
H1C	0.2114	0.6005	0.2482	0.048*	
C2	0.3672 (2)	0.4571 (3)	0.16737 (14)	0.0222 (4)	
C3	0.3316 (2)	0.3269 (2)	0.01393 (13)	0.0211 (3)	
C4	0.5089 (2)	0.2949 (2)	0.06414 (13)	0.0221 (4)	
C5	0.6677 (3)	0.2144 (3)	0.02784 (15)	0.0267 (4)	
C6	0.2221 (2)	0.2795 (3)	−0.09127 (14)	0.0241 (4)	
N1	0.24525 (19)	0.4288 (2)	0.07763 (11)	0.0216 (3)	
N2	0.5278 (2)	0.3781 (2)	0.16036 (11)	0.0226 (3)	
H2A	0.6266	0.3796	0.2086	0.027*	
O1	0.06229 (18)	0.3460 (2)	−0.11265 (10)	0.0328 (3)	
O2	0.29058 (19)	0.1816 (2)	−0.15194 (10)	0.0325 (3)	
O3	0.6306 (2)	0.1289 (2)	−0.06309 (11)	0.0362 (4)	
H3A	0.5102	0.1358	−0.0972	0.054*	
O4	0.8232 (2)	0.2331 (2)	0.07777 (12)	0.0396 (4)	

### Atomic displacement parameters (Å^2^)

**Table:**

	*U*^11^	*U*^22^	*U*^33^	*U*^12^	*U*^13^	*U*^23^
Cu1	0.01512 (18)	0.0374 (2)	0.02196 (19)	0.00490 (12)	−0.00433 (12)	−0.00595 (12)
C1	0.0263 (10)	0.0449 (12)	0.0231 (9)	0.0015 (9)	−0.0008 (7)	−0.0072 (8)
C2	0.0190 (8)	0.0268 (9)	0.0190 (8)	−0.0015 (7)	−0.0004 (6)	0.0029 (7)
C3	0.0172 (8)	0.0242 (9)	0.0205 (8)	−0.0010 (6)	0.0000 (6)	0.0001 (6)
C4	0.0189 (8)	0.0240 (9)	0.0217 (8)	0.0008 (7)	−0.0001 (6)	0.0029 (7)
C5	0.0210 (8)	0.0291 (10)	0.0294 (9)	0.0036 (7)	0.0034 (7)	0.0054 (7)
C6	0.0201 (8)	0.0304 (10)	0.0196 (8)	−0.0010 (7)	−0.0013 (6)	−0.0023 (7)
N1	0.0165 (7)	0.0275 (8)	0.0190 (7)	0.0003 (6)	−0.0013 (5)	−0.0017 (6)
N2	0.0172 (7)	0.0294 (8)	0.0184 (7)	0.0003 (6)	−0.0038 (5)	0.0025 (6)
O1	0.0206 (7)	0.0470 (9)	0.0263 (7)	0.0062 (6)	−0.0065 (5)	−0.0103 (6)
O2	0.0252 (7)	0.0441 (8)	0.0256 (7)	0.0036 (6)	−0.0010 (5)	−0.0113 (6)
O3	0.0273 (7)	0.0465 (9)	0.0342 (8)	0.0079 (6)	0.0047 (6)	−0.0077 (6)
O4	0.0196 (7)	0.0569 (10)	0.0390 (8)	0.0073 (7)	−0.0025 (6)	0.0007 (7)

### Geometric parameters (Å, °)

**Table:**

Cu1—N1	1.9633 (15)	C3—N1	1.370 (2)
Cu1—N1^i^	1.9633 (15)	C3—C6	1.478 (2)
Cu1—O1	1.9830 (14)	C4—N2	1.372 (2)
Cu1—O1^i^	1.9830 (14)	C4—C5	1.475 (3)
C1—C2	1.478 (3)	C5—O4	1.208 (2)
C1—H1A	0.9600	C5—O3	1.318 (2)
C1—H1B	0.9600	C6—O2	1.252 (2)
C1—H1C	0.9600	C6—O1	1.262 (2)
C2—N1	1.335 (2)	N2—H2A	0.8600
C2—N2	1.346 (2)	O3—H3A	0.9117
C3—C4	1.363 (2)		
			
N1—Cu1—N1^i^	180.0	C3—C4—N2	105.31 (15)
N1—Cu1—O1	83.56 (6)	C3—C4—C5	132.21 (17)
N1^i^—Cu1—O1	96.44 (6)	N2—C4—C5	121.95 (16)
N1—Cu1—O1^i^	96.44 (6)	O4—C5—O3	122.69 (18)
N1^i^—Cu1—O1^i^	83.56 (6)	O4—C5—C4	120.88 (18)
O1—Cu1—O1^i^	180.00 (5)	O3—C5—C4	116.37 (16)
C2—C1—H1A	109.5	O2—C6—O1	125.06 (16)
C2—C1—H1B	109.5	O2—C6—C3	119.96 (16)
H1A—C1—H1B	109.5	O1—C6—C3	114.98 (16)
C2—C1—H1C	109.5	C2—N1—C3	107.11 (14)
H1A—C1—H1C	109.5	C2—N1—Cu1	142.56 (13)
H1B—C1—H1C	109.5	C3—N1—Cu1	109.91 (11)
N1—C2—N2	108.89 (16)	C2—N2—C4	109.26 (14)
N1—C2—C1	126.93 (17)	C2—N2—H2A	125.4
N2—C2—C1	124.18 (16)	C4—N2—H2A	125.4
C4—C3—N1	109.42 (15)	C6—O1—Cu1	114.58 (11)
C4—C3—C6	133.84 (17)	C5—O3—H3A	114.3
N1—C3—C6	116.72 (15)		
			
N1—C3—C4—N2	0.6 (2)	C4—C3—N1—C2	−1.1 (2)
C6—C3—C4—N2	178.80 (19)	C6—C3—N1—C2	−179.58 (16)
N1—C3—C4—C5	−170.85 (19)	C4—C3—N1—Cu1	173.21 (12)
C6—C3—C4—C5	7.3 (4)	C6—C3—N1—Cu1	−5.3 (2)
C3—C4—C5—O4	163.5 (2)	O1—Cu1—N1—C2	175.3 (2)
N2—C4—C5—O4	−6.8 (3)	O1^i^—Cu1—N1—C2	−4.7 (2)
C3—C4—C5—O3	−13.6 (3)	O1—Cu1—N1—C3	4.36 (12)
N2—C4—C5—O3	176.04 (17)	O1^i^—Cu1—N1—C3	−175.64 (12)
C4—C3—C6—O2	4.4 (3)	N1—C2—N2—C4	−0.7 (2)
N1—C3—C6—O2	−177.58 (17)	C1—C2—N2—C4	179.41 (18)
C4—C3—C6—O1	−174.8 (2)	C3—C4—N2—C2	0.0 (2)
N1—C3—C6—O1	3.2 (3)	C5—C4—N2—C2	172.59 (17)
N2—C2—N1—C3	1.1 (2)	O2—C6—O1—Cu1	−178.45 (16)
C1—C2—N1—C3	−179.04 (19)	C3—C6—O1—Cu1	0.7 (2)
N2—C2—N1—Cu1	−170.06 (15)	N1—Cu1—O1—C6	−2.89 (14)
C1—C2—N1—Cu1	9.8 (4)	N1^i^—Cu1—O1—C6	177.11 (14)

Symmetry codes: (i) −*x*, −*y*+1, −*z*.

### Hydrogen-bond geometry (Å, °)

**Table:**

*D*—H···*A*	*D*—H	H···*A*	*D*···*A*	*D*—H···*A*
N2—H2A···O2^ii^	0.86	2.01	2.822 (2)	157
C1—H1A···O4^iii^	0.96	2.49	3.217 (2)	132
C1—H1B···O3^iv^	0.96	2.58	3.285 (3)	130
C1—H1C···O1^i^	0.96	2.43	3.258 (3)	144
O3—H3A···O2	0.91	1.67	2.576 (2)	171

Symmetry codes: (ii) *x*+1/2, −*y*+1/2, *z*+1/2; (iii) −*x*+3/2, *y*+1/2, −*z*+1/2; (iv) *x*−1/2, −*y*+1/2, *z*+1/2; (i) −*x*, −*y*+1, −*z*.
